# Efficacy and Safety of Dorocontin^®^ versus Sustac^®^ in the Treatment of Stable Angina Pectoris: A Randomized, Double-Blind Comparative Trial

**DOI:** 10.3797/scipharm.1406-16

**Published:** 2014-08-16

**Authors:** Yunes Panahi, Bahram Pishgoo, Yahya Dadjou, Manouchehr Mehdirad, Sara Saffar Soflaei, Amirhossein Sahebkar

**Affiliations:** ^1^ Chemical Injuries Research Center, Baqiyatallah University of Medical Sciences, Tehran, Iran.; ^2^ Cardiovascular Research Center, Baqiyatallah University of Medical Sciences, Tehran, Iran.; ^3^ Department of Cardiology, Baqiyatallah University of Medical Sciences, Tehran, Iran.; ^4^ Neurogenic Inflammation Research Center, Department of Modern Sciences and Technologies, Mashhad University of Medical Sciences, Mashhad, Iran.; ^5^ Biotechnology Research Center, Mashhad University of Medical Sciences, Mashhad, Iran.; ^6^ Cardiometabolic Research Centre, Royal Perth Hospital, School of Medicine and Pharmacology, University of Western Australia, Perth, Australia.

**Keywords:** Angina Pectoris, Nitrate, Glyceryl trinitrate, Generic, Randomized controlled trial

## Abstract

Background: Development of generic drugs has numerous benefits in terms of cost-efficiency and availability. Slow-release nitroglycerin is a vasodilator drug commonly prescribed for patients with angina pectoris. Objective: The objective of this study was to compare the efficacy and safety of generic slow-release nitroglycerin (Dorocontin^®^) with that of the innovator brand (Sustac^®^) in patients with stable angina pectoris. Methods: In this randomized, double-blind comparative trial, 110 patients were allocated to Dorocontin^®^ (n=67) or Sustac^® ^(n=43) at a dose of 6.4 mg TID, for a total period of two months. Maximum endurable MET (metabolic equivalent of task), MPI (myocardial perfusion imaging), along with changes in the ECG and biomarkers of renal (serum creatinine, BUN) and hepatic (AST, ALT, and ALP) function, lipid profile (total cholesterol, LDL-C, HDL-C, VLDL-C, and triglycerides), electrolytes (Na^+^ and K^+^), CBC-diff (RBC, WBC, Plt, Hb, Hct, MCV, MCH, MCHC, and RDW), and FBS were assessed at the baseline and at the end of the trial. The frequency of adverse events during the course of the trial was also recorded. Results: Apart from a significantly greater reduction in maximum ST depression in the Sustac^®^ versus the Dorocontin^®^ group (p=0.03), none of the functional (MET, MPI, and ECG) and paraclinical (renal function, hepatic function, lipid profile, electrolytes, and FBS) parameters significantly differed between the study groups. The mean Hb (p=0.035), Hct (p=0.045), and MCH (p=0.032) were decreased by the end of the trial in the Sustac^®^, but not in the Dorocontin^®^ group, whilst there was no change in other CBC-diff parameters. Reported adverse events were not serious and included headache, vertigo, gastrointestinal upset, and orthostatic hypotension. The frequency of these adverse events was comparable between the study groups. Conclusion: The findings of the present trial showed comparable efficacy and safety of the generic and innovator products of slow-release nitroglycerin in the management of stable angina pectoris.

## Introduction

Coronary artery disease (CAD) is the major cause of mortality and morbidity in both developing and developed countries, and is responsible for approximately 20% of total deaths worldwide [1]. Some of the most important risk factors of CAD are consumption of a high fat diet, smoking, obesity, hypertension, and type 2 diabetes mellitus. The underlying cause of CAD is atherosclerosis, which is a complex process initiated by endothelial dysfunction and subsequent atheromatous plaque formation [2]. Unstable atherosclerotic plaques are susceptible to rupture and cause arterial thrombosis and occlusion [1]. Depending on the percentage of coronary artery narrowing, clinical features of CAD can differ from an asymptomatic disease to acute myocardial infarction. Nitrates are among the most widely prescribed medications for the treatment of ischemic heart diseases including CAD. Medical effects of nitroglycerin (glyceryl trinitrate) involve the bioconversion to nitric oxide (NO) by the enzyme aldehyde dehydrogenase. NO promotes systemic vasodilatation and epicardial coronary artery distention, thereby reducing heart oxygen demands and increasing coronary artery blood flow [3]. In spite of its efficacy, the use of nitrates is accompanied by a number of adverse events such as tachycardia, orthostatic hypotension, and pulsatile headache; all resulting from vasodilatation [4].

Slow-release nitroglycerin is the most widely-used nitrate in clinical practice. Although efficacious, the treatment-associated cost is a limitation of the use of slow-release niacin, as maintenance therapy with this medication is usually long-term, and in a considerable proportion of patients, lifelong. The introduction of efficacious generic products would increase patients’ access to the medication and, on the other hand, reduce treatment-associated costs [5–8]. The present study aimed to compare the efficacy and safety of a generic slow-release nitroglycerine product, Dorocontin^®^ (manufactured by Dorsa Darou Pharmaceutical Co, Iran), versus the innovator product Sustac^®^ (Krka Pharmaceutical Co., Slovenia) in patients with stable angina pectoris.

## Material and Methods

This study was designed as a randomized, double-blind clinical trial conducted between 2010 and 2011 in the Cardiology Clinic at the Baqiyatallah Hospital, Tehran, Iran. Inclusion criteria were age >40 years and a diagnosis of stable angina pectoris, according to clinical manifestations, an exercise test, and paraclinical findings by a board-certified cardiologist. Exclusion criteria were the presence of diabetes, renal failure, anemia, liver disease, malignancy, pathologic changes in the electrocardiogram (ECG), elevation of cardiac enzymes, and intake of other vasodilator drugs such as calcium channel blockers.

One-hundred and forty subjects met the inclusion criteria and were randomly allocated to either Dorocontin^®^ (*n* = 70) or Sustac^®^ (*n* = 70) for a period of two months. The administered dose of either of the medications was 6.4 mg three times per day. The demographic and anthropometric characteristics of the subjects were recorded in questionnaires. All subjects underwent an exercise test at the baseline and at the end of the trial in order to determine maximum endurable MET (metabolic equivalent of task) and MPI (myocardial perfusion imaging). Electrocardiograms were also assessed for pathological changes such as ST depression and inverted T. Overnight-fasted blood samples were taken from all individuals at the baseline and at the end of the trial. After isolation of the serum, the concentrations of total cholesterol, low-density lipoprotein cholesterol (LDL-C), high-density lipoprotein cholesterol (HDL-C), very low-density lipoprotein cholesterol (VLDL-C), triglycerides (TG), glucose (FBS), aspartate amino-transferase (AST), alanine aminotransferase (ALT), alkaline phosphatase (ALP), creatinine (Cr), and blood urea nitrogen (BUN) were determined. A complete blood count with differentials (CBC-diff) comprising red blood cell (RBC) count, white blood cell (WBC) count, platelet (Plt) count, mean corpuscular volume (MCV), mean corpuscular hemoglobin (MCH), mean corpuscular hemoglobin concentration (MCHC), and red cell distribution width (RDW) was also determined for each subject at the baseline and trial end. Patients were asked to report any adverse events experienced during the course of the trial. The study protocol was approved by the Institutional Ethics Committee and written informed consent was obtained from all participants.

Statistical analyses were performed using SPSS software, version 15. Within-group comparisons (pre vs. post) were made using the paired samples *t*-test (for normally distributed data) or the Wilcoxon signed-ranks test (for non-normally distributed data). Changes in each evaluated parameter during the course of the study was compared between the Dorocontin^®^ and Sustac^®^ groups using the independent samples *t*-test (for normally distributed data) or the Mann-Whitney U test (for non-normally distributed data). Categorical variables were compared using the Chi-square or Fisher’s exact test. A two-sided *p*-value of < 0.05 was considered to be statistically significant in all analyses.

## Results

**Fig. 1.**
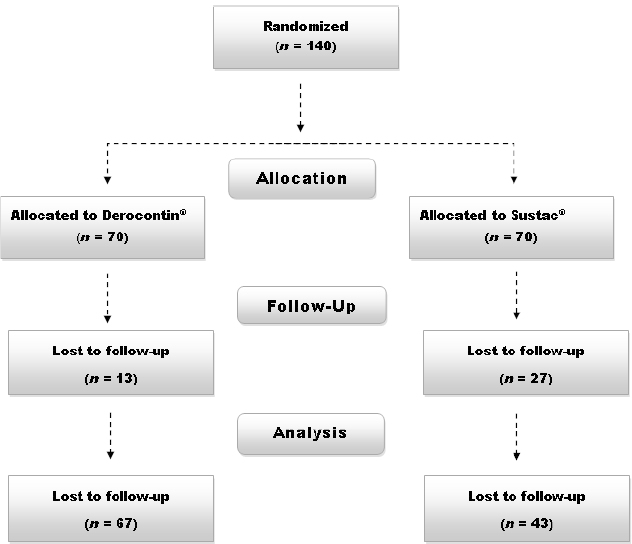
Flow diagram of the trial

Out of the 140 individuals who initially entered the trial, 110 completed the study: 67 in the Dorocontin^®^ and 43 in the Sustac^®^ group (Figure 1). Drop-outs were due to the loss of follow-up and thus, did not return for the second visit and blood sampling. Data obtained from these completers were included in the final analysis.

### Demographic Characteristics

The groups were comparable regarding age, gender, body mass index (BMI), and duration of disease diagnosis. The frequency of previous admission because of ischemic heart disease was significantly higher in the Dorocontin^®^ (71.4%) versus Sustac^®^ (40.4%) group. However, the history of admission to the coronary care unit (CCU), and frequencies of diabetes mellitus, hypertension, and chronic respiratory diseases did not differ between the study groups. Demographic characteristics of the study participants are summarized in Table 1.

### Cardiac Factors

As shown in Table 2, maximum ST depression during the exercise test did not change significantly in either of the study groups. However, the magnitude of the reduction was significantly greater in the Sustac^®^ versus Dorocontin^®^ group (*p* = 0.03). The values of heart rate, MET, and MPI did not significantly change in either of the groups by the end of the trial, nor was any difference between the groups in terms of changes in each parameter. Frequencies of ECG abnormalities including ST depression and inverted T were reduced in the Dorocontin^®^ group in a borderline significant manner, but there was no change in the Sustac^®^ group. Changes in the frequencies of ECG abnormalities were comparable between the study groups (Table 2).

**Tab. 1.** Demographic characteristics of the study groups

Variable	Dorocontin® (*n* = 67)	Sustac® (*n* = 43)	Overall (*n* = 110)	*p*-value**
Age (years)	58.77 (8.49)	59.02 (9.08)	58.88 (8.71)	0.883
BMI (kg/m2)	27.34 (4.32)	28.20 (3.79)	27.69 (4.11)	0.335
Duration of disease diagnosis (years)	6.70 (6.24)	4.99 (6.5)	5.87 (5.72)	0.143
Male	35 (55.6%)	28 (59.6%)	63 (57.3%)	0.701
DM	19 (30.2%)	19 (40.4%)	38 (34.5%)	0.313
HTN	32 (50.8%)	20 (42.6%)	52 (47.3%)	0.443
Chronic respiratory disease	4 (6.3%)	8 (17.0%)	12 (10.9%)	0.120
History of admission	45 (71.4%)	29 (40.4%)	64 (58.2%)	0.002
History of CCU admission	33 (52.4%)	22 (46.8%)	55 (50.0%)	0.700

Values are expressed as the median (SD) or number (%). BMI: body mass index; DM: diabetes mellitus; HTN: hypertension; CCU: coronary care unit.

**Tab. 2.** Cardiac function before and after intervention in the study groups

Test	Factor	Group	Pre-trial		Post-trial		*p*-Value^a^	*p*-Value^b^
			*Median*	*SD*	*Median*	*SD*		
Exercise Test	Maximum ST Depression (mV)	Dorocontin^®^	0.048	0.19	0.047	0.19	0.970	0.03
		Sustac®	0.063	0.203	0.038	0.197	0.507	
	MET (× basal energy consumption)	Dorocontin®	10.98	2.51	11.24	2.30	0.414	0.828
		Sustac®	20.65	2.62	10.77	2.50	0.819	
	Heart rate (n)	Dorocontin®	144.24	28.57	146.20	21.31	0.564	0.419
		Sustac®	139.69	26.13	136.78	26.58	0.571	
ECG	Inverted T (mV)	Dorocontin®	0.72	0.5	0.65	0.52	0.083	0.605
		Sustac®	0.50	0.50	0.57	0.50	0.317	
	ST Depression (mV)	Dorocontin®	0.72	0.50	0.65	0.52	0.083	0.681
		Sustac®	0.46	0.50	0.57	0.50	0.180	
MPI	MPI Index	Dorocontin®	0.72	0.90	0.63	0.80	0.564	0.717
		Sustac®	0.60	0.54	0.60	0.54	0.999	

^a^ Within-group comparison;

^b^ Between-group comparison; ECG…Electrocardiography; MPI…Myocardial Perfusion Imaging; MET…Metabolic Equivalent of Task.

### Clinical Chemistry

Clinical chemistry parameters including serum lipid profile (total cholesterol, LDL-C, HDL-C, VLDL-C, and triglycerides), biomarkers of hepatic (ALT, AST, and ALP) and renal function (BUN and Cr), electrolytes (Na^+^ and K^+^), and FBS remained statistically unchanged by the end of the trial in both studied groups. Likewise, the between-group comparison of the magnitude of changes did not reveal any significant difference in the above-mentioned parameters (Table 3).

**Tab. 3.** Cell blood count with differentials before and after intervention in the study groups

Variable	Groups	Pre-trial		Post-trial		*p*-Value^a^	*p*-Value^b^
		*Median*	*SD*	*Median*	*SD*		
RBC (10^6^/mm^3^)	Dorocontin®	4.73	0.52	4.75	0.55	0.718	0.906
	Sustac®	4-90	0.55	4.91	0.86	0.917	
Hb (g/dL)	Dorocontin®	13.66	1.64	13.75	1.33	0.496	0.035
	Sustac®	14.32	1.95	13.98	1.92	0.019	
Hct (%)	Dorocontin®	40.76	4.42	40.83	3.87	0.825	0.045
	Sustac®	42.37	5.56	41.39	5.39	0.014	
Plt (10^3^/mm^3^)	Dorocontin®	219.34	54.65	215.78	60.71	0.440	0.145
	Sustac®	197.18	37.71	206.48	53.13	0.276	
WBC (10^3^/mm^3^)	Dorocontin®	7.94	8.63	8.12	10.69	0.627	0.702
	Sustac®	6.71	1.39	6.69	1.25	0.935	
MCV (fL)	Dorocontin®	86.72	5.39	85.51	5.40	0.067	0.226
	Sustac®	86.64	5.19	84.26	5.56	0.002	
MCH (pg)	Dorocontin®	33.65	1.26	33.82	1.21	0.426	0.762
	Sustac®	33.74	1.17	33.81	1.25	0.790	
RBW (%)	Dorocontin®	19.23	12.79	19.36	12.95	0.88	0.551
	Sustac®	13.42	1.65	12.84	1.23	0.148	

^a^ Within-group comparison;

^b^ Between-group comparison; RBC…red blood cell; WBC…white blood cell; Plt…platelet; Hb…hemoglobin; Hct…hematocrit; MCV…mean corpuscular volume; MCH…mean corpuscular hemoglobin; MCHC…mean corpuscular hemoglobin concentration; RDW…red cell distribution width.

### CBC-diff

Levels of Hb (*p*=0.035), Hct (*p*=0.045), and MCH (*p*=0.032) were significantly decreased by the end of study in the Sustac^®^, but not Dorocontin^®^ group, yielding a significant between-group difference. MCV values were decreased in the Sustac^®^ group, but remained unaltered in the Dorocontin^®^ group, yielding a comparable magnitude of changes between the groups. Other hematologic parameters including RB, WBC, and platelet counts, MCHC, and RDW were not changed in any of the study groups (Table 4).

### Adverse Events

No serious adverse event was reported from the study medications. Reported adverse events were headache (*n* = 17 in the Dorocontin^®^ and *n* = 9 in the Sustac^®^ group), vertigo with tinnitus (*n* = 9 in the Dorocontin^®^ and *n* = 6 in the Sustac^®^ group), gastrointestinal upset (*n* = 4 in the Dorocontin^®^ and *n* = 1 in the Sustac^®^ group), and orthostatic hypotension (*n* = 3 in the Dorocontin^®^ group). Systolic and diastolic blood pressures in the Dorocontin^®^ group changed from 133 mmHg and 97 mmHg to 129 mmHg and 100 mmHg, respectively. In the Sustac^®^ group, the systolic and diastolic blood pressures changed from 134 mmHg and 97 mmHg to 133 mmHg and 98 mmHg, respectively.

**Tab. 4.** Comparison of clinical chemistry parameters between the study groups

Variable	Groups	Pre-trial		Post-trial		*p*-Value^a^	*p*-Value^b^
		*Median*	*SD*	*Median*	*SD*		
FBS (mg/dL)	Dorocontin®	132.03	63.83	123.62	63.67	0.222	0.352
	Sustac®	127.06	50.98	128.63	46.28	0.840	
Na^+^ (meq/L)	Dorocontin®	140.95	2.79	141.10	2.82	0.736	0.113
	Sustac®	139.50	3.01	140.95	3.28	0.069	
K^+^ (meq/L)	Dorocontin®	4.12	0.40	4.19	0.43	0.315	0.851
	Sustac®	4.27	0.41	4.32	0.45	0.593	
BUN (mg/dL))	Dorocontin®	18.57	7.99	18.21	7.22	0.574	0.974
	Sustac®	17.38	5.85	16.99	5.58	0.623	
Creatinine (mg/dL)	Dorocontin®	1.27	0.97	1.21	0.74	0.274	0.160
	Sustac®	1.29	1.0	1.33	0.99	0.148	
SGOT (U/L)	Dorocontin®	28.14	15.26	27.60	14.93	0.688	0.592
	Sustac®	27.75	12.27	28.39	9.68	0.710	
AIP (U/L)	Dorocontin®	209.37	99.75	204.64	90.36	0.283	0.579
	Sustac®	203.00	64.94	194.03	63.16	0.182	
Total cholesterol (mg/dL)	Dorocontin®	163.56	40.69	161.84	31.98	0.718	0.861
	Sustac®	162.39	45.41	159.03	40.10	0.706	
LDL-C (mg/dL)	Dorocontin®	84.69	25.90	86.71	27.10	0.466	0.223
	Sustac®	94.44	40.36	87.48	36.57	0.309	
HDL-C (mg/dL)	Dorocontin®	43.15	11.71	43.60	11.18	0.712	0.602
	Sustac®	41.33	10.56	42.86	10.62	0.383	
VDL-C (mg/dL)	Dorocontin®	38.22	34.01	36.89	30.74	0.660	0.557
	Sustac®	31.21	9.93	32.32	11.00	0.575	
Triglycerides (mg/dL)	Dorocontin®	164.40	121.61	160.46	119.45	0.642	0.693
	Sustac®	149.44	50.19	150.89	65.26	0.890	

^a^ Within-group comparison;

^b^ Between-group comparison; FBS…fasting blood sugar; BUN…blood urea nitrogen; SGOT…serum glutamate oxaloacetate transaminase; SGPT…serum glutamate pyruvate transaminase; Alp…alkaline phosphatase; LDL-C…low-density lipoprotein cholesterol; HDL-C…high-density lipoprotein cholesterol; VLDL-C…very low-density lipoprotein cholesterol.

## Discussion

In the pharmaceutical industry, generics are defined as economical medications with comparable safety and therapeutic efficacy to brand-name innovator products [9]. Generic drugs are on average around 20–90% cheaper than their originator counterparts [10]. An analysis of data from the 1997–2000 Medical Expenditure Panel Survey Household Component in the US indicated that using a generic instead of a brand-name drug can save around 78 million dollars per year for adults older than 65 years [5]. Owing to the cost-efficiency and wider availability of generic drugs compared with their brand-name counterparts, many countries have adopted a generic prescription policy e.g. UK, Norway, Belgium, and Iran (since 1980) [6–8, 11, 12].

Nitrates are one of the most commonly in-use vasodilators that are routinely prescribed for the treatment of angina pectoris [13]. Due to systemic vasodilatation, nitrates decrease venous return to the heart and myocardial stretch, therefore reducing myocardial oxygen demands. Moreover, nitrates increase coronary blood flow of epicardial coronary arteries [3]. Slow-release nitroglycerin is available in Iran in two forms: the generic Iran-made product (Dorocontin^®^) and the brand-name product (Sustac^®^). The objective of this study was to compare the efficacy and safety of these two products in patients with stable angina pectoris in the setting of a randomized double-blind comparative trial.

The results indicated that there was no significant difference between the study groups with respect to pathological changes in ECG, heart rate, MET, and MPI during the exercise test. Also, the assessment of the clinical chemistry data revealed that none of the paraclinical data including lipid profile parameters, hepatic and renal function biomarkers, serum electrolytes, and glucose were significantly different between the study groups. Dorocontin^®^ had not only no adverse impact on hematologic parameters, but was also superior to Sustac^®^, as it did not cause any decrease in Hb, Hct, and MCH.

With respect to side effects, no serious adverse event occurred in the study groups. Reported adverse reactions were mild and included headache, vertigo, gastrointestinal discomfort, and orthostatic hypotension. Frequencies of these adverse events were comparable between the study groups. Headache was the most common side effect of nitroglycerin in both groups. This is consistent with the results of a systematic review and meta-analysis in 2011, in which a high prevalence of headaches (52%) was reported in patients receiving nitrates [14]. However, the frequency of headaches in both groups in the present trial was much lower (25% in the Dorocontin^®^ and 20% in the Sustac^®^ group) which favors the tolerability of both studied drugs.

A limitation of the current trial was the high rate of drop-outs. Nevertheless, drop-outs are unlikely to have overestimated the efficacy and safety of the generic drug Dorocontin^®^, as the rate of drop-out was higher with Sustac^®^ compared with Dorocontin^®^. Since the main aim of this study was to show the non-inferiority of Dorocontin^®^ to Sustac^®^, lower drop-out with Dorocontin^®^ could be regarded as a positive finding showing higher compliance and possibly, acceptable tolerability of this drug. As far as safety is concerned, the frequency of adverse events during the course of the trial was not significantly different between the study groups, and there was no report of serious events.

In summary, the present randomized, controlled trial provided evidence as to the equivalent efficacy and safety of the generic product Dorocontin^®^ and the innovator brand product Sustac in patients with stable angina pectoris. According to the manufacturer’s report, substitution of Sustac^®^ with generic Dorocontin^®^ would save about 7 million dollars annually. Therefore, the lower cost of this generic product may encourage its use in routine clinical practice. Whilst the present data is encouraging, further research is required to assess the efficacy and safety of Dorocontin^®^ in the long-term.
